# Deposits in the Human Body

**Published:** 1847-03

**Authors:** 


*Deposits in the Human Body.*—The following scheme includes the various substances capable of occurring as deposits in the human body:

1. Protein compounds.
2. Fats: *a*, cholesterin; *b*, margarin and margaric acid; *c*, olein; *d*, fatty granules of uncertain composition.
3. Uric acid and urates.
4. Salts of lime: *a*, oxalate of lime; *b*, basic phosphate of lime; *c*, carbonate of lime.
5. Ammoniaco-magnesian phosphate.
6. Sulphuret of iron.
7. Bile-pigment.
8. Silica.
9. Various substances of easy solubility, deposited in consequence of evaporation, etc., as chloride of sodium, phosphate of soda, etc.

From one or more of these constituents are formed all the concretions occurring in the human body. These are arranged by Vogel into

1. Such as are produced from the secreted fluids.
2. Such as are formed in the parenchyma of organs.—*Ibid.*

